# Severe Peripartum Cardiomyopathy With Complete Functional Recovery: The Role of Multimodality Imaging and Optimized Medical Therapy

**DOI:** 10.7759/cureus.109938

**Published:** 2026-05-30

**Authors:** Cesar Baltodano Dangla, Christopher Kaleb Romero Ríos, Juan Cristobal Mendoza, Mario Canales, Enamaria Villalta Garcia, Tania M Gamez, César Jeovany Urbina Maliaños, Andres Zamora, Byron Larios Alemán, Jasson E Rostran Ruíz, Winston S Castillo, Lidia Saénz

**Affiliations:** 1 Cardiology, Hospital Militar Escuela "Dr. Alejandro Dávila Bolaños", Managua, NIC; 2 School of Medicine, Hospital Militar Escuela "Dr. Alejandro Dávila Bolaños", Managua, NIC; 3 Radiology, Hospital Vivian Pellas, Managua, NIC; 4 Radiology, Hospital Militar Escuela "Dr. Alejandro Dávila Bolaños", Managua, NIC; 5 Oncology, Hospital Militar Escuela "Dr. Alejandro Dávila Bolaños", Managua, NIC; 6 Medicine, Hospital Militar Escuela "Dr. Alejandro Dávila Bolaños", Managua, NIC; 7 Research, Hospital Militar Escuela "Dr. Alejandro Dávila Bolaños", Managua, NIC

**Keywords:** biventricular dysfunction, cardiac magnetic resonance, echocardiography, global longitudinal strain, guideline-directed medical therapy, intracardiac thrombosis, left ventricular systolic dysfunction, peripartum cardiomyopathy, postpartum heart failure, ventricular function recovery

## Abstract

Peripartum cardiomyopathy (PPCM) is a life-threatening, idiopathic form of heart failure with a highly variable recovery trajectory. While normalization of the left ventricular ejection fraction (LVEF) is frequently achieved, identifying residual myocardial injury and subclinical dysfunction remains a significant diagnostic challenge. A 25-year-old woman presented with acute heart failure seven weeks postpartum, following a pregnancy complicated by preeclampsia. Initial multimodality imaging revealed severe biventricular dysfunction (LVEF 14%, right ventricular ejection fraction 17%), intracavitary apical thrombi at the left ventricular apex, and a peak NT-proBNP of 12,241 pg/mL. Comprehensive management included therapeutic anticoagulation (initially with bemiparin), early guideline-directed medical therapy, and a prolonged 12-week course of bromocriptine. At the 18-month follow-up, the patient achieved complete clinical recovery (New York Heart Association (NYHA) class I) with normalization of LVEF. However, advanced imaging identified persistent subclinical impairment, evidenced by an abnormal global longitudinal strain (averaging -15.0%) and focal myocardial fibrosis (3% of total mass) on cardiac magnetic resonance. This case illustrates that clinical and conventional echocardiographic recovery in PPCM may mask persistent subclinical myocardial dysfunction. It emphasizes the necessity of multimodality imaging, specifically speckle-tracking strain analysis and tissue characterization, to accurately define the true spectrum of myocardial recovery and guide long-term risk stratification.

## Introduction

Peripartum cardiomyopathy (PPCM) is an idiopathic, life-threatening form of heart failure characterized by left ventricular systolic dysfunction (left ventricular ejection fraction (LVEF) <45%) occurring toward the end of pregnancy or within the first five months postpartum [1]. While its global incidence varies widely, the underlying pathophysiology is increasingly linked to an antiangiogenic environment, driven by the oxidative cleavage of prolactin into a cardiotoxic 16-kDa fragment [2]. The clinical presentation is highly heterogeneous, ranging from mild dyspnea to cardiogenic shock. In severe cases (LVEF <30%), profound hemodynamic stasis combined with pregnancy-related hypercoagulability significantly elevates the risk of life-threatening complications, particularly intracavitary thrombosis, which requires prompt recognition and targeted management [3,4].

While transthoracic echocardiography (TTE) and natriuretic peptides remain the cornerstone of initial diagnosis, conventional LVEF measurements often fail to capture the full spectrum of myocardial recovery [5]. Clinical improvement and LVEF normalization can mask persistent subclinical structural remodeling. Consequently, multimodality imaging incorporating cardiac magnetic resonance (CMR) for tissue characterization and speckle-tracking echocardiography for myocardial deformation analysis has become essential for refined longitudinal monitoring [6,7].

We report a case of severe postpartum-onset PPCM complicated by biventricular failure and apical thrombi. This case highlights the critical role of multimodality imaging in identifying residual subclinical dysfunction despite conventional LVEF recovery and illustrates the successful implementation of the "BOARD" (Bromocriptine, Optimized HF therapy, Anticoagulation, Vasorelaxation, Diuretics) protocol, achieving excellent clinical outcomes [8].

## Case presentation

A 25-year-old woman (body mass index 24 kg/m²) with no previous surgical history, toxic habits, or family history of cardiomyopathy experienced a first pregnancy complicated by preeclampsia at 35 weeks of gestation. The hypertensive disorder was managed conservatively with labetalol and nifedipine until she underwent an uncomplicated vaginal delivery at term.

Approximately 50 days postpartum, the patient developed progressive dyspnea and fatigue, initially attributed to a nonspecific viral illness. As her functional status rapidly deteriorated to New York Heart Association (NYHA) class IV, she was admitted to the hospital. Physical examination revealed jugular venous distension, bilateral pulmonary crackles, and peripheral edema. The initial electrocardiogram demonstrated sinus tachycardia (120 beats per minute) with nonspecific ST-segment and T-wave changes, without evidence of acute ischemia. Laboratory testing showed a marked elevation of N-terminal pro-B-type natriuretic peptide (NT-proBNP) at 2,879 pg/mL.

Initial TTE revealed dilated left-sided cardiac chambers, severe biventricular systolic dysfunction (estimated LVEF of 19%), eccentric remodeling, and severe functional mitral regurgitation. The patient required a total hospitalization of seven days. During the acute phase, CMR imaging was performed, confirming severe biventricular failure with an LVEF of 14% and right ventricular ejection fraction (RVEF) of 17%. CMR also identified multiple intracavitary thrombi at the left ventricular apex (measuring up to 18 mm), minimal non-ischemic subendocardial late gadolinium enhancement (LGE), and a mild pericardial effusion (Figures 1-3). To definitively exclude other potential etiologies of acute biventricular failure, a transesophageal echocardiogram (TEE) was performed, which confirmed intact interatrial and interventricular septa without evidence of vegetations or congenital shunts. A 24-hour Holter monitor ruled out complex ventricular arrhythmias.

**Figure 1:**
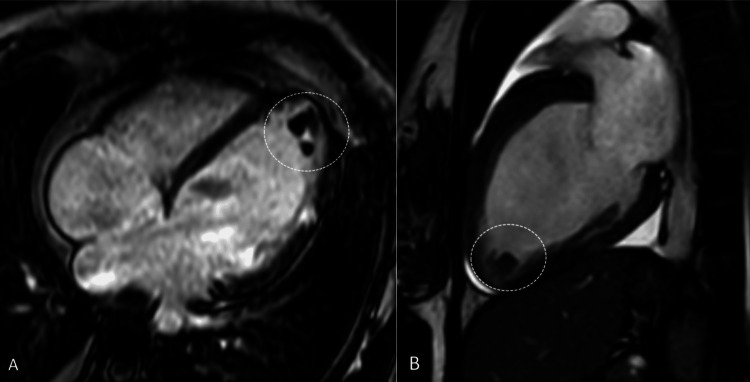
Cardiac magnetic resonance A: Long cardiac axis, four-chamber view; B: Long cardiac axis, two-chamber view. This T2-weighted image shows several images in the left ventricle that condition signal void artifact (dotted circle) and that measure between 7 and 18 mm, which correspond to intracavitary thrombi. Furthermore, the left ventricle (best shown in B) is mildly enlarged, which constitutes a sign of severe systolic dysfunction.

**Figure 2:**
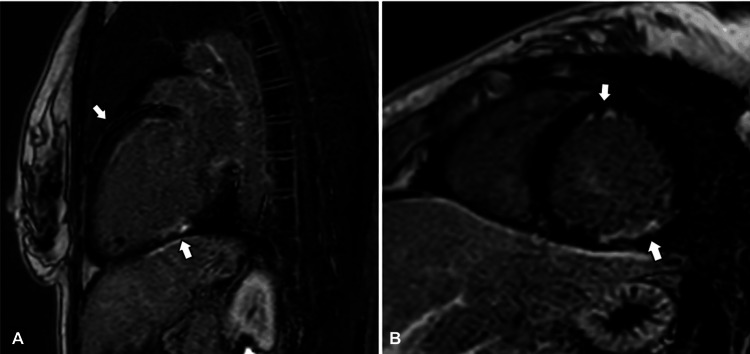
T1-weighted image, late gadolinium enhancement (LGE) A: Long cardiac axis, two-chamber view. B: Short cardiac axis, two-chamber view. After administration of gadolinium-based contrast medium, non-transmural subendocardial enhancements (white arrows) of less than 25% were observed in the anterior mid-segment (7) and greater than 75% in the mid-inferior segment (10) according to the standardized American Heart Association (AHA) 17-segment model [9].

**Figure 3:**
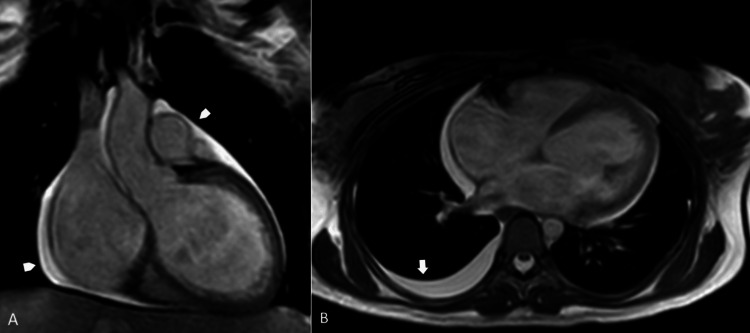
T2-weighted cardiac magnetic resonance imaging A: Coronal view; B: Axial view. T2-weighted cardiac magnetic resonance imaging demonstrates a mild pericardial effusion (pentagon arrow), with an estimated 11-mm separation between the pericardial layers. A moderate right pleural effusion (white arrow) is also observed in panel B.

Following hemodynamic stabilization with intravenous loop diuretics, the patient was started on therapeutic anticoagulation due to the apical thrombi. Initial management consisted of subcutaneous bemiparin 7,500 IU daily (therapeutic dose). After the high-risk acute phase, she was transitioned to oral anticoagulation with rivaroxaban 20 mg daily for long-term outpatient follow-up. Guideline-directed medical therapy (GDMT) for heart failure was aggressively initiated. Crucially, lactation was pharmacologically suppressed, allowing for the early introduction of an angiotensin receptor-neprilysin inhibitor (sacubitril/valsartan 25 mg twice daily, later uptitrated to 50 mg), alongside bisoprolol 5 mg daily, dapagliflozin 10 mg daily, and spironolactone 25 mg daily. To address the pathophysiological role of prolactin, the patient completed a standard 12-week course of bromocriptine (initiated at 2.5 mg twice daily for two weeks, followed by 2.5 mg daily).

The patient was discharged for close outpatient follow-up. While her clinical congestion resolved, her NT-proBNP levels transiently peaked at 12,241 pg/mL two months post-discharge before initiating a progressive decline. Serial echocardiograms over the following months demonstrated a restrictive diastolic filling pattern with slowly improving systolic function.

Approximately nine months after disease onset, repeat CMR demonstrated significant partial recovery (LVEF 43.4%), complete resolution of the intracavitary apical thrombi, and a persistent but small area of focal myocardial fibrosis representing approximately 3% of the total myocardial mass (Figure 4). NT-proBNP levels had decreased to 200 pg/mL.

**Figure 4:**
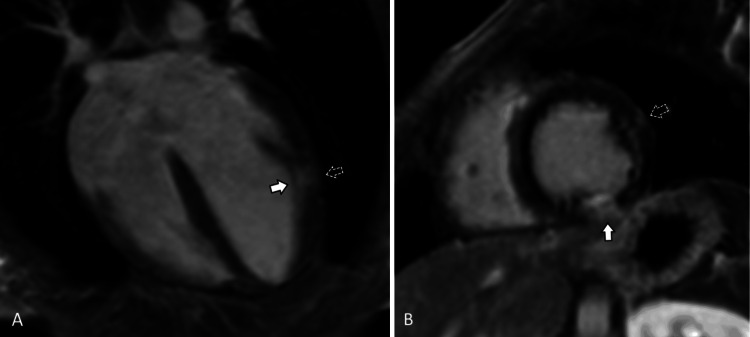
Nine-month follow-up late gadolinium enhancement (LGE) cardiac magnetic resonance imaging A: Long-axis four-chamber view; B: Short-axis two-chamber view. The late gadolinium enhancement (LGE) sequence demonstrates non-transmural subendocardial enhancement (white arrow) involving approximately 25% of the mid-inferior segment (10), as well as focal intramyocardial enhancement (white-dotted arrow) in the mid-inferolateral (11) and mid-anterolateral (12) segments of the lateral wall. These findings correspond to a fibrotic area involving approximately 3% of the total myocardial mass.

Correlative TTE during this intermediate recovery phase (Figure 5) showed a left ventricle with normal dimensions and wall thickness, without dilatation or hypertrophy. The LVEF calculated by the Simpson biplane method was 41.4%. Crucially, advanced myocardial deformation analysis revealed a reduced global longitudinal strain (GLS) of −14.2%, confirming global systolic dysfunction despite the preserved ventricular geometry.

**Figure 5:**
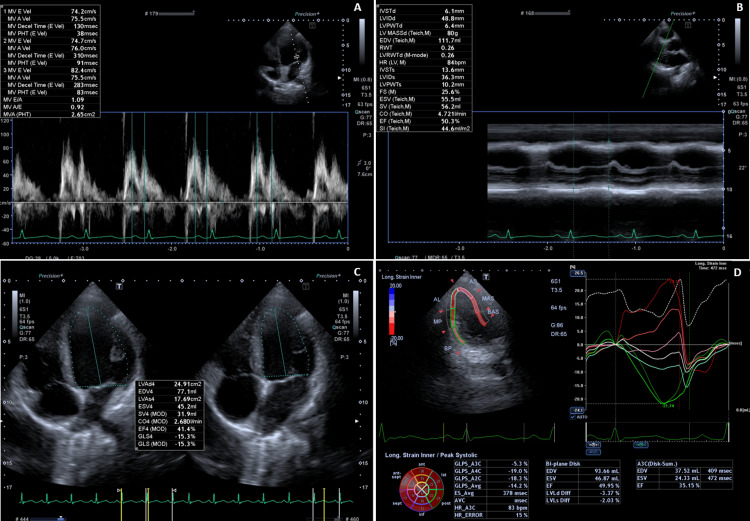
Transthoracic echocardiography assessment of left ventricular structure and function A: Pulsed-wave Doppler of transmitral inflow demonstrating preserved diastolic function without evidence of elevated left ventricular filling pressures. B: Parasternal long-axis M-mode showing normal left ventricular dimensions and wall thickness. C: Left ventricular ejection fraction calculated by the Simpson method in the apical four-chamber view (41.4%). D: Myocardial deformation analysis revealing reduced global longitudinal strain (−14.2%) with an estimated ejection fraction of 39%, indicating persistent subclinical systolic dysfunction.

During subsequent long-term follow-up at approximately 18 months, the patient achieved complete clinical recovery (NYHA class I) and remained entirely asymptomatic. Serial TTE demonstrated sustained structural normalization with LVEF further improving and stabilizing in the near-normal to mildly reduced range (46%-60% across serial evaluations). However, speckle-tracking echocardiography persistently revealed a reduced GLS averaging -15.0% to -15.6%, confirming the presence of residual subclinical systolic dysfunction despite profound clinical improvement. Given the favorable clinical evolution, device-based therapies (implantable cardioverter-defibrillator) were deemed unnecessary. The patient continues long-term maintenance on an optimized regimen consisting of sacubitril/valsartan 50 mg twice daily, bisoprolol 5 mg daily, dapagliflozin 10 mg daily, and spironolactone 25 mg daily.

## Discussion

The diagnosis of peripartum cardiomyopathy is fundamentally one of exclusion. It is defined by the onset of heart failure with reduced ejection fraction toward the end of pregnancy or in the months following delivery, in the absence of another identifiable cause [1]. In the present case, the diagnosis was established by strictly following the criteria of the Heart Failure Association of the European Society of Cardiology (ESC), which require the systematic exclusion of alternative etiologies. The use of transesophageal echocardiography was crucial to confirm intact interatrial and interventricular septa and the absence of vegetations, effectively ruling out undiagnosed congenital anomalies or infectious endocarditis. Similarly, CMR imaging allowed us to rule out infiltrative diseases and massive myocardial necrosis, solidifying PPCM as the primary pathology. The association with preeclampsia during the preceding pregnancy further supports the pathophysiological link between PPCM and an antiangiogenic milieu, specifically the elevation of soluble fms-like tyrosine kinase-1 (sFlt-1), which promotes intense endothelial and myocardial injury in susceptible individuals [10].

This case exemplifies the remarkable potential for reverse remodeling in PPCM, even when initial parameters suggest a dismal prognosis. Data from the Investigations of Pregnancy-Associated Cardiomyopathy (IPAC) registry demonstrate that an initial LVEF <30% and biventricular involvement are powerful predictors of delayed or incomplete myocardial recovery [5]. Our patient presented with profound biventricular dysfunction (LVEF 14%, RVEF 17%), functional atrioventricular valvular regurgitation, and intracavitary apical thrombi. The biomarker profile correlated with clinical severity, marked by an initial NT-proBNP of 2,879 pg/mL and a delayed peak of 12,241 pg/mL. This transient elevation, occurring despite clinical decongestion, reflects the pathophysiological discordance between resolved volume overload and ongoing transmural mechanical stress driven by biventricular dysfunction [11]. Furthermore, while recent cohorts establish that a baseline NT-proBNP ≥900 pg/mL is a strong predictor of failure to recover left ventricular function [12], our patient achieved complete structural normalization. Aligning with contemporary data, it was the subsequent downward NT-proBNP trajectory, rather than the initial peak, that accurately predicted the definitive systolic recovery observed in this case [13,14].

Given the relevance of multimodality imaging, the integration of CMR and advanced echocardiography was pivotal. While CMR confirmed a non-ischemic dilated phenotype, the detection of a focal subendocardial LGE pattern warranted careful consideration of an ischemic etiology. However, ischemic cardiomyopathy was excluded due to the profound discordance between the minimal total burden of fibrosis (approximately 3%) and the extreme severity of the biventricular dysfunction. In severe PPCM, such isolated LGE foci can occasionally be attributed to microvascular ischemia or silent micro-embolization originating from concurrent apical thrombi [15].

A critical finding was the persistence of subclinical dysfunction despite LVEF recovery. While conventional echocardiography demonstrated structural normalization (LVEF 46-60%), myocardial deformation analysis revealed a persistently reduced GLS averaging -15.0%. This highlights that the recovery of ejection fraction may mask persistent alterations in myocardial mechanics. Contemporary evidence suggests that impaired GLS in patients with recovered LVEF is associated with a higher risk of adverse events, justifying rigorous long-term follow-up [7].

Pharmacological management involved the early optimization of GDMT. Extrapolating from the most recent universal guidelines for heart failure [16], the use of sacubitril/valsartan and an SGLT2 inhibitor in the acute phase was made possible by the pharmacological suppression of lactation. This eliminated the primary contraindication for these agents and allowed for aggressive reverse remodeling. The addition of bromocriptine as part of the "BOARD" protocol is based on the inhibition of the cardiotoxic 16-kDa prolactin fragment [2,9]. While the ESC supports the use of bromocriptine based on randomized trials [17], the AHA/ACC guidelines maintain a more cautious stance (Class IIb recommendation), citing the need for larger multicenter trials [16].

Finally, regarding sudden cardiac death prevention, definitive device-based therapies were deferred to allow for spontaneous recovery [1,5]. From a reproductive counseling perspective, the patient was informed that her residual subclinical dysfunction (GLS impairment) places her at a high risk of relapse in subsequent pregnancies, necessitating multidisciplinary preconception evaluation [18].

## Conclusions

This case provides critical clinical pearls for the management of severe PPCM. First, definitive diagnosis in the postpartum period mandates the rigorous clinical exclusion of delayed hypertensive heart failure secondary to preeclampsia and systemic coagulopathies. Second, the normalization of conventional ejection fraction does not equate to complete myocardial healing; advanced deformation analysis (GLS) and tissue characterization are essential to unmask persistent subclinical dysfunction. Finally, while early implementation of comprehensive GDMT can drive remarkable structural recovery even in extreme biventricular failure, the persistence of underlying myocardial impairment underscores that true recovery is often incomplete, dictating the need for vigilant long-term follow-up and strict multidisciplinary preconception counseling.
